# More than 9,000,000 Unique Genes in Human Gut Bacterial Community: Estimating Gene Numbers Inside a Human Body

**DOI:** 10.1371/journal.pone.0006074

**Published:** 2009-06-29

**Authors:** Xing Yang, Lu Xie, Yixue Li, Chaochun Wei

**Affiliations:** 1 Shanghai Center for Bioinformation Technology, Shanghai, China; 2 School of Life Science and Technology, Tongji University, Shanghai, China; 3 Department of Bioinformatics and Biostatistics, School of Life Sciences and Biotechnology, Shanghai Jiao Tong University, Shanghai, China; 4 Bioinformation Center, Shanghai Institutes for Biological Sciences, Chinese Academy of Sciences, Shanghai, China; 5 Lab of Molecular Microbial Ecology and Ecogenomics, School of Life Sciences and Biotechnology, Shanghai Jiao Tong University, Shanghai, China; Charité-Universitätsmedizin Berlin, Germany

## Abstract

**Background:**

Estimating the number of genes in human genome has been long an important problem in computational biology. With the new conception of considering human as a super-organism, it is also interesting to estimate the number of genes in this human super-organism.

**Principal Findings:**

We presented our estimation of gene numbers in the human gut bacterial community, the largest microbial community inside the human super-organism. We got 552,700 unique genes from 202 complete human gut bacteria genomes. Then, a novel gene counting model was built to check the total number of genes by combining culture-independent sequence data and those complete genomes. 16S rRNAs were used to construct a three-level tree and different counting methods were introduced for the three levels: strain-to-species, species-to-genus, and genus-and-up. The model estimates that the total number of genes is about 9,000,000 after those with identity percentage of 97% or up were merged.

**Conclusion:**

By combining completed genomes currently available and culture-independent sequencing data, we built a model to estimate the number of genes in human gut bacterial community. The total number of genes is estimated to be about 9 million. Although this number is huge, we believe it is underestimated. This is an initial step to tackle this gene counting problem for the human super-organism. It will still be an open problem in the near future.

The list of genomes used in this paper can be found in the supplementary table.

## Introduction

Estimating the number of genes in human genome has been one of the most fundamental problems in computational biology. The number of genes estimated for human genome dropped from more than 100,000 [1] to 60,000 [2], 40,000 [3], and 30,000 after the draft human genome came out in 2001 [4], then to the current estimation of about 23,000 [5]. This number is not much larger than 17,000, the number of genes in *C.elegans* [6], a model organism about 1 mm in length and about 1000 cells in total. The methods used to estimate gene numbers include transcript-based methods, CpG island counting methods and ultimately, gene prediction methods. Transcript-based methods contain cDNA counting, EST clustering and Refseq gene counting, etc. Although the accurate number of genes in a human genome is still not determined, the scale of the gene number has been set to be about 20,000.

In the other hand, a human body contains not only the human genome. Microbes inhabit ubiquitously in or on our human body, such as lung, skin, oral cavity, etc. A new concept is to consider human as a super-organism containing those microbes in or on human body as well [7]. There are more than 100 trillion bacterial cells in human gut, which are about 10 times more than cells in human itself [8]. Those bacteria can help digest food and harvest nutrition and energy that otherwise cannot be collected by the human body directly [9]–[11], i.e., human has obtained many genes needed for itself though these genes did not evolve in human genome.

With the progress of molecular biotechnology and data accumulation, our understanding about gene and disease is under a revolution: diseases are not only associated with genes in human genome but also related to genomes from environment around and inside human body. A notable example of what environmental genome changes can result in is human gut bacterial community. The change of human gut bacterial community is associated with obesity [9], diabetes [12], hypertension [13], and so on. Therefore gut bacteria has become one of the hot research areas especially for those researches about chronic and metabolism related diseases [14]. Also, it is becoming an important research direction for finding drug target genes using human gene networks [15]. Drug target genes can also be identified by studying similar gene networks containing mixture of both human genes and genes from human gut bacteria [16]. Further studies in these directions require that we understand the gene composition of the human gut microbe community. Estimating the number of genes in it is one of the most important steps to understand the scale of the problem that we are dealing with.

Based on the diversity of gut microbes and the average number of genes contained in a microbe genome, the number of genes in human gut microbiota was guessed to be 100 times greater than that of our human genome [17]. Since the scale of total number of genes in a human genome is about 20,000. This makes the guess of human gut microbiota genes to be at least 2,000,000. With the current available 16S rRNA sequences and hundreds of complete microbe genomes, it is possible to give a better estimation on the total number of genes by combining these two data sources. However, there is no similar research done before as the authors are aware of. The most similar research was done for pan-genomes, which contains all unique genes in a species with similar genes from different strains merged. In a pan-genome study, people check a “core-genome” with genes present in all strains, a “dispensable genome” with genes present in two or more genomes and a “unique genome” with genes unique to each strain. Gene numbers have been counted for pan-genomes for some species [18], and models have been constructed to represent the total number of genes in a species from the number of genes in the strains that belong to this species. However, there is no similar method applied to estimate gene numbers in a microbe community such as the human gut bacterial community.

In this paper, we present a model to estimate the number of protein-coding genes in human gut bacterial community, the largest microbe community in or on human body. We estimate the number of genes in human gut, both the number of overall genes and the number of core genes, which are conserved across multiple microbes.

While the update and further accumulation of genomic and metagenomic data may result in a more reliable and accurate estimation, how genes are defined in the counting can affect the estimation result by an order of magnitude. Therefore, a clear definition of genes should be given before we set off the journey of gene counting. In the upcoming section, we will define the terms we use in the paper,

### Terms definition

In this section, we define the following six terms used in this paper: genes, orthologs, paralogs, core genome, pan-genome and genome combination. Their meaning may be different in other literatures.

The term “gene” in this paper stands for “protein coding” genes. This precludes other functional genes, such as RNAs.

Orthologs are genes found in different species but originated from a common ancestor and thus, often have similar functions. Here, we call two genes from different genomes “orthologs” if their sequence similarity reaches a certain threshold.

Paralogs are genes generated from gene duplication in the same genome and do not always have the same function. In this paper, two genes from the same genomes are called “paralogs” if their similarity reaches a certain threshold.

Core genome is the set of genes which are common to every selected genome. Usually the term “core genome” is used when no less than two genomes were considered, while we extended its usage to one genome. The core genome for a single genome can be viewed as the set of none duplicated genes, or the “de-paralogged” genome.

Pan-genome is the whole set of genes in a number of genomes, including core genes which are shared by all genomes, partially shared genes which can be found in some genomes but absent from the others, and strain-specific genes. The concept of “pan-genome” typically is used at genus or species level. We extend it to higher taxa. Also, the pan-genome for one genome is allowed in our study to denote the set of non-redundant genes of that genome.

The term “combination” of genomes is used in our counting of total genes and core genes. Two similarity cutoffs were set in the combination: the paralog and ortholog similarity thresholds. A similarity of 0.90 can't distinguish genes with similarity of 0.95. As a result, after the combination, genes with similarity above predetermined threshold can't be distinguished and are recognized as the same gene. The union set of combination is the pan-genome while the intersection describes the core genome. A higher similarity resolution will generate a larger size of pan-genome and a lower similarity resolution will yield a larger size of core-genome, accordingly.

## Result

### Estimation of overall number of genes in human gut bacterial community

#### 202 gut bacterial genomes

**Figure 1:**
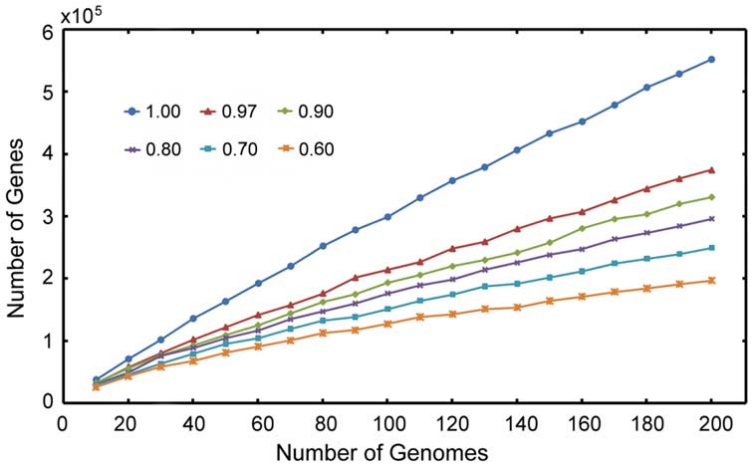
Pan-genome of 202 gut bacteria. The nodes denote the total gene number after genome combination, each of which is the average value of 30 times of sampling. Sampling size scales from ten to two hundred genomes, with a step size of ten. Thresholds for circle, triangle, diamond, cross, square and asterisk markers are 1.00, 0.97, 0.90, 0.80, 0.70 and 0.60, respectively. Paralogs and orthologs used the same thresholds in this combination. Accessions and information for the two hundred and two genomes used in the figure can be found in Table S1 (see lines marked as “1” in the “Figure” column).

Two hundred and two human gut bacterial genomes were selected (see Methods and Materials part for details) from Genome Project database and downloaded from Genbank [19]. The corresponding protein coding sequences of these genomes were extracted from NCBI annotation table and similarities among these sequences were obtained by running all-against-all WU-Blast blastn (http://blast.wustl.edu/). Genomes were combined according to their protein coding sequence similarity percentages to get their pan-genome. The results of combination at similarity cutoff from 0.60 to 1.00 are shown in Figure 1. The same threshold for paralogs (genes from the same genome) and orthologs (genes from different genomes) was used in this combination. Accessions and information for the 202 genomes can be found in Table S1 (the column “Figures” will tell which genomes were used in which figures).

#### Pan-Genome analysis for strain-to-species level and species-to-genus level

**Figure 2:**
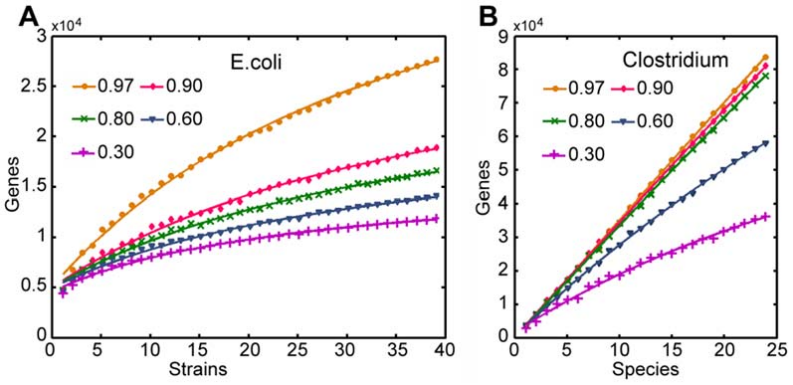
Pan-genome of 39 *E. coli* strains and 24 *Clostridium* species. Figure A shows the pan-genome of 39 *E. coli* strains at different similarity cutoff. Figure B shows the pan-genome of 24 different *Clostridium* species. Similarity percentage cutoff in both A and B for the circle, diamond, cross, triangle and plus sign are 0.97, 0.90, 0.80, 0.60 and 0.30, respectively. The same cutoff was used for paralogs and orthologs. Each node is the average value a number of times of sampling. Sampling times can be found in Table 2. The corresponding lines are generated by least square curving fitting of the nodes with function F(n) = [a−b/(x+c)]*Avg, coefficients of which are available in Table 1. Accessions and information for 39 *E.coli* and 24 *Clostridium* genomes used in the figure can be found in Table S1 (see the “Figure” column, lines marked as “2”).

Thirty nine *E. coli* genomes and twenty four *Clostridium* genomes which represent twenty four different *Clostridium* species were downloaded from Genbank. A same process applied to the 202 genomes in Figure 1 was performed on the 39 *E. coli* and 24 *Clostridium* genomes to generate the colored nodes in Figure 2. The lines in Figure 2 were derived by least square curve fitting the colored nodes which are the average values of the combination results for a number of sampling. The information about the 39 *E. coli* and 24 *Clostridium* studied can be found in Figure S1. Some of the 39 *E. coli* and 24 *Clostridium* genomes incorporated in our study are not human gut bacteria and were used here to improve the reliability of our model. We used the function
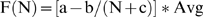
(1)for the fitting, assuming that there is a limited gene number for one species or genus. *Avg stands for the average gene numbers for all genomes after redundant genes with similarity higher than a threshold were merged.

The coefficients for the curve fit functions generated using least squares method at different thresholds for 39 *E. coli* strains and 24 *Clostridium* species are shown in Table 1.

**Table 1:** Function coefficients for lines in Fig. 2 for 39 *E. coli* and 24 *Clostridium*.

Category	Similarity	Avg Genes	Coefficients*
*E. coli*	0.97	4756	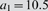 , 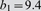 , 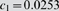
	0.9	4707	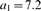 , 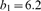 , 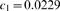
	0.8	4674	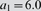 , 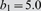 , 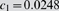
	0.6	4567	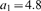 , 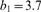 , 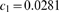
	0.3	4393	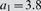 , 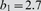 , 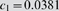
*Clostridium*	0.97	3524	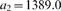 , 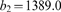 , 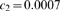
	0.9	3501	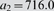 , 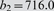 , 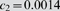
	0.8	3483	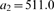 , 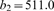 , 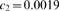
	0.6	3387	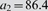 , 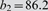 , 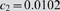
	0.3	2685	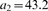 , 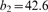 , 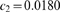

This table provides information for the 39 *E. coli* and 24 *Clostridium* analyzed in Figure 2. The nodes in Figure 2A and Figure 2B are the average value for 30 and 20 times of sampling, respectively. *Avg genes are the average gene numbers for all genomes after they are de-paralogged (combined with itself at certain cutoff). Coefficients* were obtained by least square curve fitting function F(n) = [a−b/(n+c)]*Avg. a_1_, b_1_ and c_1_ are for 39 *E. coli* in Figure 2A while a_2_, b_2_ and c_2_ are for 24 *Clostridium* in Figure 2B. Accessions and other information for the genomes can be found in Table S1.

**Figure 3:**
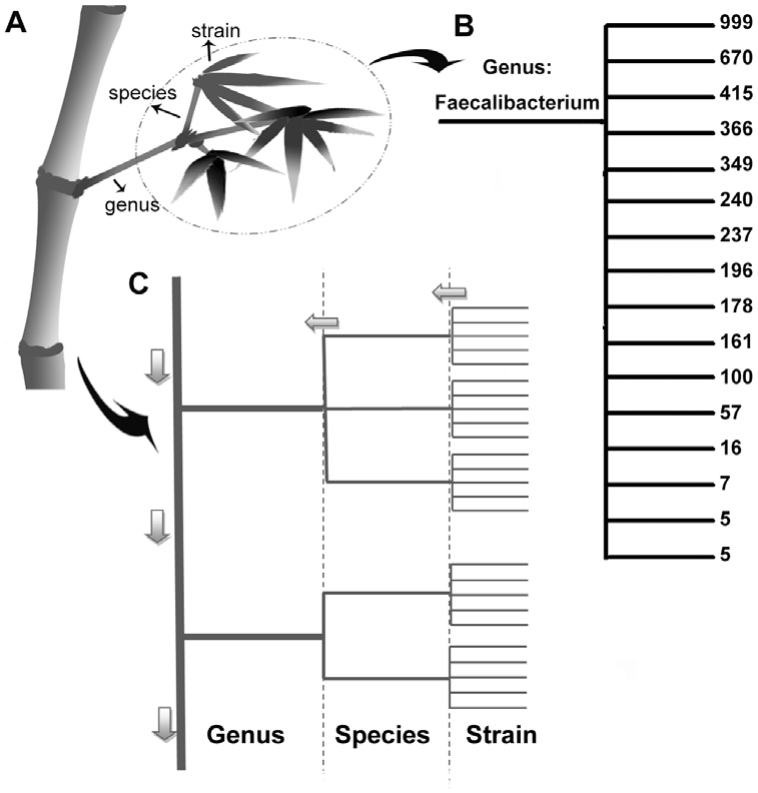
The gene-counting model: a bamboo-like-tree structure of human gut bacterial community. Figure A shows how the gut bacterial community is visualized as a bamboo. B gives an example how the genus *Faecalibacterium* with 4,105 strains is clustered into. First, a tree was built from the 16S rRNA distance matrix of the 4,105 strains using UPGMA method, and then suspicious branches which consist less than 0.1% of the population were trimmed off. C shows the general pipeline of our counting model. Genes were first counted among strains within a species, and then among species within a genus. The total gene number of the community can be obtained by adding genes in every genus together.

In contrast to genome within one species, the 24 *Clostridium* genomes from 24 different species showed only a slight sharing of genes at high similarity cutoff. The poor performance of combination at species level avoids us of the attempt at higher levels, such as similarity among different families, and intuited our bamboo-like tree (Figure 3) construction for the whole gut bacterial community. A number of foliages share the same stem. The stem itself shares with other stems the same branch that connects them to the cane (Figure 3A). Imagine a genus of bacteria as a branch, the stems can be considered as species in that genus, and the foliages of a stem stand for different strains in a species.

#### The gene counting model

Figure 3C shows how our counting was carried out. The three-level tree for each genus was built using Unweighted Pair Group Method with Arithmetic Mean (UPGMA) method, a simple clustering method in which the distance between any 2 clusters is defined by the mean distance between elements of each cluster. The trees were then connected disregarding their higher lineages. The number of genes was counted within each species first (with function (1)). These numbers together with their corresponding rank numbers of the species then went to the next stop at the species-to-genus joint where their contribution for the final result of our counting is calculated by function (1) with the first level results as inputs. Gene number for each genus can be calculated and then the whole gene number for gut bacterial community can be obtained by summing gene numbers in all genera together.

#### Pan-Genome estimation for human gut bacteria

The two requisite elements of our counting are incorporating of citizens who inhabit our guts and selecting of models which are fit to represent all these citizens. The Human Microbiome Project provides us hundreds of sequenced bacterial genomes, from which we can pick our models if the number of sequenced genomes within a species or genus reaches a considerable value (we modeled from 39 *E. coli* strains and 24 *Clostridium* species). As for the whole citizen, we resort to culture-independent data to get a glimpse of their identities.

Composition of a bacterial community usually is studied in culture-based methods. However, the majority of gut citizens are “inculturable”. High-throughput sequencing technologies and culture-independent methods enable us to sequence metagenomes that cannot be cultured in labs. Particularly, directly sequencing of 16S rRNA genes can provide us culture-independent approaches to identify the existence of the otherwise inapproachable majority.

**Figure 4:**
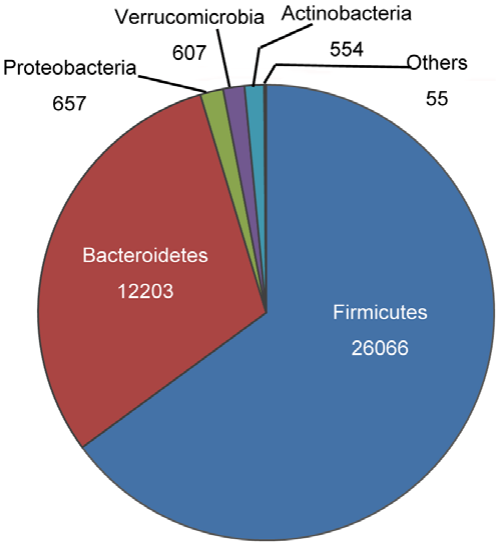
Gut bacteria composition. The pie chart shows the distribution of gut bacteria obtained by searching RDP browser. Others are: *Spirochaetes*, *Fusobacteria*, *Deferribacteres*, *Cyanobacteria*, *Planctomycetes*, *Lentisphaerae*, *TM7* and *Tenericutes*. Of the thirteen phyla, th*e Firmicutes* and *Bacteroidetes* occupy 65% and 30% of the pie, respectively, while the rest eleven phyla take up 5%.

In our study, the composition of human gut bacteria was obtained by searching RDP's browser [20], [21], which, up to date, has 836,814 16S rRNAs, more than 90% of which are of good quality [22]. The search options of our work were limited to sequences no shorter than 1200 bp and with good quality to preclude poor qualified sequences as much as possible. The search returned a result of 40,180 sequences which belong to 13 phyla (Figure 4).

Bacteria from 8 of these 13 phyla were spotted in human gut microbiota in 2005 [17]. It was pointed out that of the 8 phyla, Bacteroidetes and Firmicutes constitute the dominant part while the rest comprise only a slight proportion, which tallies the result reported by Eckburg et al [23].

The distance matrixes for gut bacteria were downloaded from RDP browser at genus level. Distance matrix for each genus is then used to cluster these 16S rRNAs into different groups (species) at the cutoff of 0.02, which is a commonly used threshold for species [17]. An example of how the Genus *Faecalibacterium* is clustered is shown in Figure 3B. The tree was built from the 16S rRNA distance matrix of the 4,105 strains using UPGMA method, and suspicious branches which consist less than 0.1% of the population were trimmed off. The remained species branches were then rearranged by the number of strains they contain.

Although our search of RDP browser returned 40,180 sequences, sequences marked “unclassified” in RDP browser were excluded in our analysis since our trees were built from strains in the same genus.

**Figure 5:**
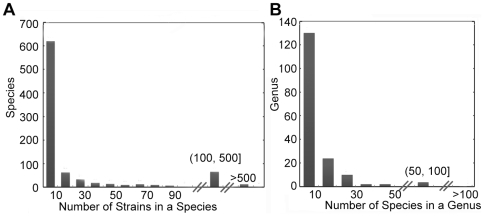
*Cl*uster result of human gut bacteria based on 16S rRNA sequences. 16S rRNA sequences of 164 genera were downloaded from RDP browser. More than 94% of the 164 genera have less than 30 species and more than 84% of the 826 species have less than 40 strains.

We tested the fidelity of our strain-to-species model and species-to-genus model in the 39 E. coli strains and 24 *Clostridium* species, respectively. Then the distribution of strain number in a species and the species number in a genus was checked. The gene number may be underestimated if the strain number is greater than 39 or the species number is larger than 24. Figure 5 shows the distribution of species and strains based on 16S rRNA sequences from RDP database. More than 94% of the 164 genera have less than 30 species, and of all the 826 species, >84% contain no more than 40 strains. Therefore, the model we build for strain-to-species and species-to-genus fits majority of the cases.

The final step of our counting is to apply our models to every strain in every species with the model validated in the 39 *E. coli* genomes, and use the resulted numbers as inputs to the species-to-genus model validated in 24 *Clostridium* genomes. The results of our estimation are presented in Table 2. Estimations scale from 2,932,368 at cutoff of 30% similarity to 8,988,806 at cutoff of 97%. Due to the underestimation in multiple steps, we conclude that there are more than 9,000,000 unique genes in the human gut bacterial community.

**Table 2:** Estimated total gene numbers for human gut bacterial community.

Similarity	0.97	0.90	0.80	0.60	0.30
Genes	8,988,806	6,533,896	5,799,165	4,071,772	2,932,368

The strain-to-species and species-to-genus gene counting used the same similarity at 0.97, 0.90, 0.80, 0.60 and 0.30 in the estimation. Detail of the gene counting model can be found in the gene counting model part of the Result section.

#### Core-genome analysis of genus Bacteroides and other four species

**Figure 6:**
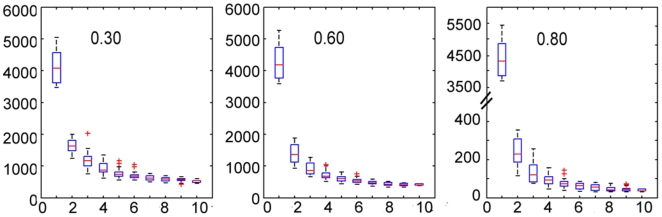
Core-genome sizes for 10 *Bacteroides* genomes at different similarity cutoffs. Each genome represents a different species. Accessions and information for the ten genomes can be found in Table S1.

Core genome denotes the set of genes which can be found in every genome, at a certain level of similarity. We studied the core genome for genus *Bacteroides*, which comprise a major portion of the human normal gut flora. Figure 6 shows the core genome sizes for ten *Bacteroides* at similarity cutoff of 0.30, 0.60 and 0.80. About 400–500 genes were found common in the 10 Bacteroides species at similarity thresholds of 0.30 and 0.60. The number dropped drastically to about 50 when similarity was set to 0.80. More than half of the 50 genes are ribosomal proteins while the rest function mainly in replication or translation.

Core genes are much more common at species level than that of genus level, as indicated by our core-genome analysis of seven *C. perfringen*s, eight *C. difficile*, nine *C. botulinum* and twenty six *E. coli* strains. At similarity cutoff from 0.30–0.90, we got approximately 1500, 2000, 2000 and 1300 core genes for *C. perfring*ens, *C. difficile*, *C. botulinum* and *E. coli*, respectively. The detail of core genes for the four species can be found in Figure S1 and Table S1.

## Materials and Methods

### Bamboo-like tree construction for gut bacterial community

126 uncorrected distance matrixes (DNADist format) were downloaded from RDP website [20], [21], representing 126 genera of human gut bacteria. 16S rRNA sequences were obtained by searching RDP browser with the default set of: both type and non-type, uncultured and isolate strains with good quality and size > = 1200 bp. The search text for gut bacterial community is: (((bacteri* OR archae*) AND human AND (GI OR gut OR colon* OR mucous OR intestin* OR fecal OR stool OR feces OR faec*)) NOT (oral OR dental OR gastritis OR nasopharyngeal OR periodontal OR urinary)) NOT (large subunit OR 15S OR 23S).

For each genus, a percent identity cutoff of 0.98 [17]was used to build a depth-2 phylogenetic tree. The uncorrected distance we used is the proportion of nucleotide sites at which two sequences compared are different, which was obtained by dividing the number of different sites by the total length of sequences compared. Thus, a similarity cutoff of 0.98 and a distance cutoff of 0.02 can be used interchangeably. The depth-2 tree for each genus was constructed using Unweighted Pair Group Method with Arithmetic Mean (UPGMA) method.

Figure 3B gives an example of how the genus *Faecalibacterium* was grouped into. The species branches were rearranged with descendant order of the strain numbers they have after the UPGMA tree building. Branches that constitute less than 0.1% of all population were trimmed off the tree to exclude suspicious sequences, for example, branches with less than 5 strains were trimmed off in Figure 3B. As a result, about 2.5% of the total 4,105 sequences of this genus were missing from the tree.

### 202 complete gut bacterial genomes

The 202 genomes in Figure 1 were obtained from NCBI Genome Project Database by search text: (bacteria OR archaea OR archaeal) AND human AND (GI OR gut OR colon* OR intestin* OR fecal OR feces OR faec* OR stool) NOT (oral OR dental OR gastritis OR nasopharyngeal OR periodontal OR urinary), and then selected from the search results whose status are “complete” or “draft assembly”. Their citizenship of human gut bacteria were identified by manually checking their corresponding dossiers in Genome Project Database. The 202 qualified genomes of gut bacteria, which represent 39 genera, were downloaded from GenBank. Details of the 202 genomes can be found in Table S1.

**Figure 7:**
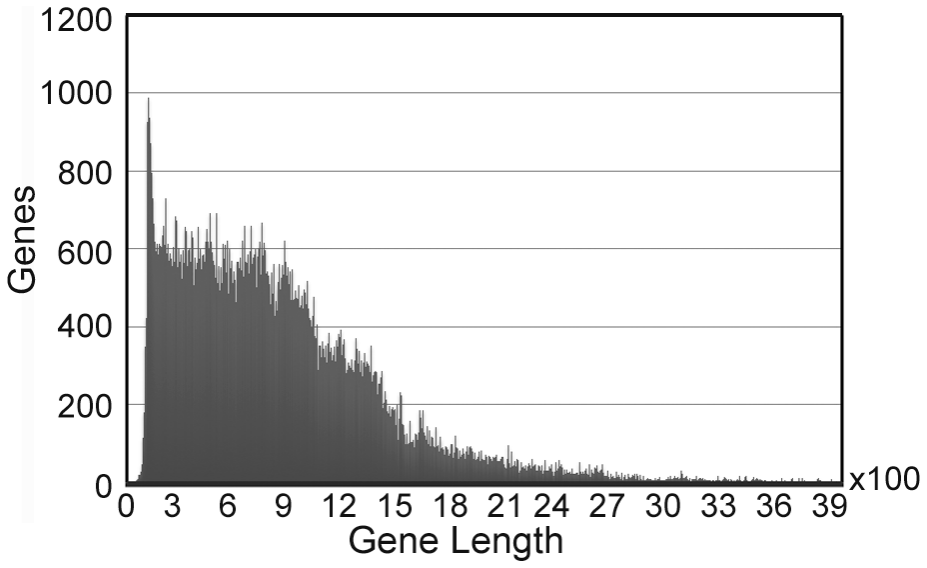
Gene length distribution for genes of 202 gut bacteria genomes. This figure shows the distribution of 202 gut bacteria genomes' gene lengths as annotated by NCBI. The genes used in this study were annotated by NCBI as protein coding sequences. Y-axis tells the number of genes with a certain length.

Protein coding sequences were downloaded from NCBI ftp were they in complete genomes or extracted from NCBI annotation file if they were from “draft assembly” genomes. NCBI annotations for bacteria were done by Prokaryotic Genomes Automatic Annotation Pipeline (PGAAP), which predicts genes using a combination of GeneMark and Glimmer. Without evidence such as existing of RNA or similarity with existing proteins to draw forth confident identification of a gene, we evaluate genes provided by NCBI by their lengths. Figure 7 shows the distribution of gene length for all genes of the 202 genomes. A dramatic jump around gene length of 105 bp was spotted and predicted genes with length shorter than or equal to 105 were considered as suspicious genes which may be from false prediction and were exempted in the consequent analysis. Due to this 105 bp cutoff, the total number of genes may be slightly underestimated.

### Calculation of similarity/identity percentage

Genes for 202 genomes were run all-against-all WU-Blast 2.0 blastn to generate a tabular output, which is a tab-delimited text file. WU-Blast options were -w 14, -wink 8, -e 10, -Q 9, -R 8. The tabular output of WU-Blast is then further used to generate the similarity percentage (or identity percentage) between each query and subject sequence by dividing the number of matches by the total length of query sequence (Nmatch/Nall). The extracted files which contain information for query and subject gene accessions and their similarity percentage are further used for in genome combination for overall genes and core genes analysis.

## Discussion

Estimating the number of genes in or on human body is an interesting problem that has a large number of audients. There are two highlights of this paper:

1. We presented a novel model to estimate the number of genes in human gut by combining culture-independent sequencing data and completed microbe genomes currently available.
2. The total number of genes is estimated to be 8,988,806 in human gut bacterial community, and we believe it is underestimated.

In a similar analysis, Tettelin et al shows that the number of additional genes to a pan-genome varies largely for different species when adding a new strain to a species. For some species, the additional number of genes by adding one new strain to the species is going to be a positive constant, which implies that there are an infinite number of genes in a microbe species [24]. In our model, similar trend is observed only at the species-to-genus level when the similarity threshold is set to 97%. At the strain-to-species level, we believe our model is more reasonable since the total number of genes is going to increase to an upper bound approximately when more strains are counted. In addition, since we estimated the parameters for different species based on the complete genomes currently available, the accuracy of the model can be improved when more complete microbe genomes are finished in the near future.

There are two potential problems in our estimation since we used all 16S rRNA sequences from rdp database with a certain quality criterion. First, whether16S rRNA is capable of grouping strains into species is still debatable. Second, the 16S rRNA sequences may come from different individuals. Therefore, the estimated number of genes reported in this paper can be counted from many individuals. In our model, the number of genes in an individual human gut can be estimated by combining meta-genomic data of this individual and correspondent completed genomes. In fact, the number of unique 16S rRNA in an individual is still unclear. In a recent research, Turnbaught et al sequenced 10,000 V6 regions of 16S rRNAs for each of 154 individuals, and found there was little overlap between the sampled fecal communities. The estimation model we presented in this paper is relatively simple. It is a very initial step to tackle this problem. Therefore, estimating the total number of genes in an individual human gut is still an open problem.

## Supporting Information

Table S1 Genomes used in this paper. Figure*2: the numbers in this column are the figures appeared in the paper or the supplementary material, take the third genome as an example, “1,2,8” indicates NC_002655 was used in the pan-genome analysis for 202 genomes, the pan-genome analysis for 39 E.coli and the core genome analysis for 26 E.coli. Genome*1: The genus name for each genome has been abbreviated to one capital. 1–39: Escherichia; 40–86: Clostridium; 87–97: Bacteroides; 98–107: Campylobacter; 108–110: Providencia; 111: Proteus; 112–116: Listeria; 117: Rickettsia; 118: Salmonella; 119–151: Salmonella enterica subsp. enterica serovar; 152: Salmonella enterica subsp; 153–155: Collinsella; 156: Victivallis; 157–160: Ruminococcus; 161–168: Bacillus; 169–175: Bifidobacterium; 176–183: Shigella; 184–185: Dorea; 186–187: Streptococcus; 188–197: Vibrio; 198–199:Tropheryma; 200: Mitsuokella; 201–203: Lactobacillus; 204: Methanosphaera; 205–207: Eubacterium; 208–209: Parabacteroides; 210–211: Yersinia; 212: Methanobrevibacter; 213: Klebsiella; 214: Akkermansia; 215: Actinomyces; 216: Faecalibacterium; 217: Enterobacter; 218: Anaerostipes; 219: Peptostreptococcus; 220: Coprococcus; 221: Alistipes; 222: Anaerotruncus; 223: Anaerofustis; 224: Roseburia.
(0.03 MB XLS)

Figure S1 Core genome for four species. Figure A, B, C and D show the core genome sizes for 7 C.perfringens, 8 C.difficile, 9 C.botulinum and 26 E.coli, respectively. Accession and information for genomes used in this analysis can be found in supplementary Table S1.
(0.35 MB TIF)
