# Supplementary figures and images for: More than 9,000,000 Unique Genes in Human Gut Bacterial Community: Estimating Gene Numbers Inside a Human Body
Source: PLoS One. 2009 Jun 29;4(6):e6074. doi: 10.1371/journal.pone.0006074 (PMC2699651; doi:10.1371/journal.pone.0006074)

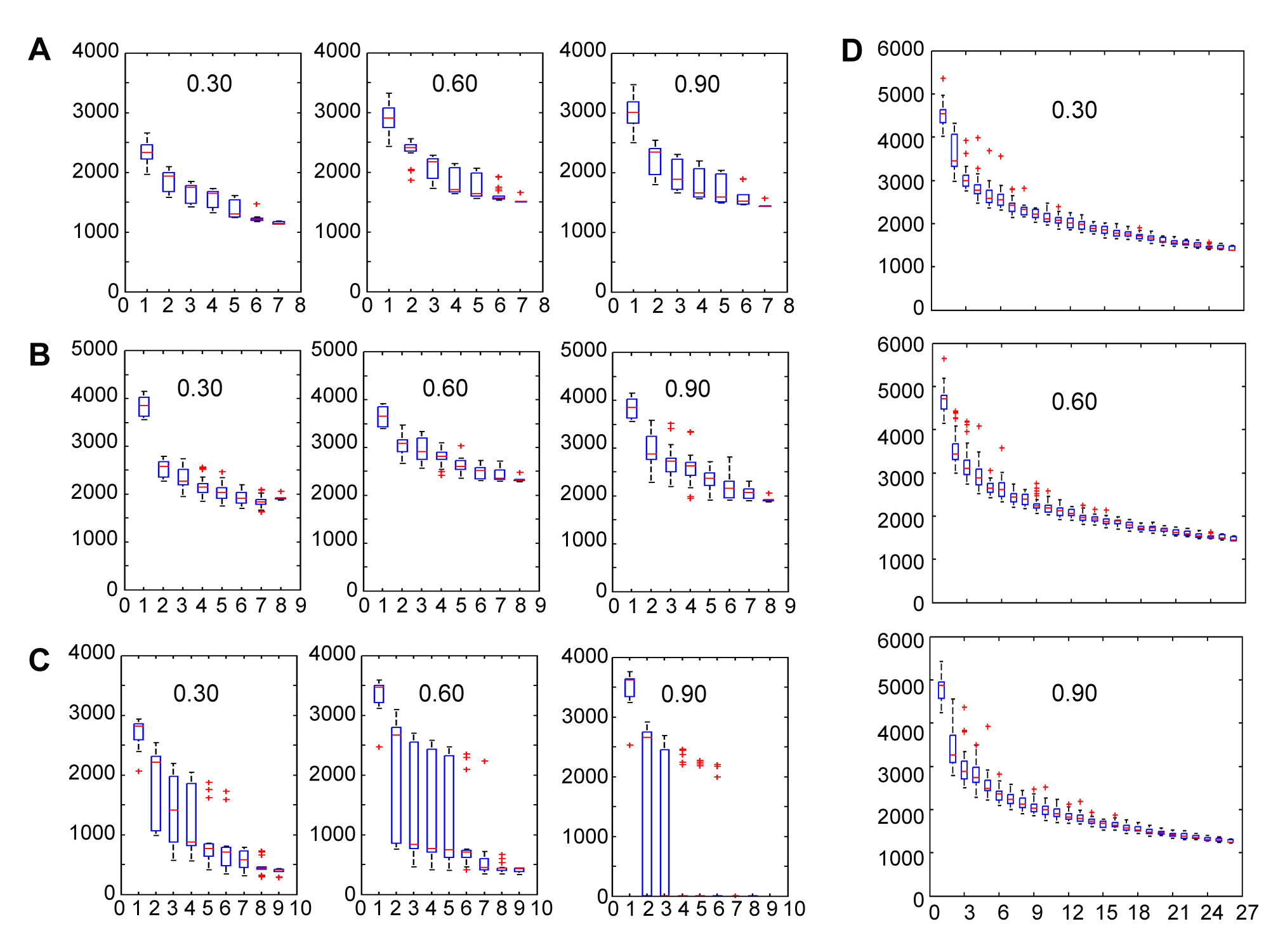

Supplement: Figure S1 — Core genome for four species. Figure A, B, C and D show the core genome sizes for 7 C.perfringens, 8 C.difficile, 9 C.botulinum and 26 E.coli, respectively. Accession and information for genomes used in this analysis can be found in supplementary Table S1. (0.35 MB TIF) [file pone.0006074.s002.tif]
